# Test-Time Optimization of Physical ISP Parameters from a Single Calibration Pair for Cross-Camera Adaptation †

**DOI:** 10.3390/s26206542

**Published:** 2026-10-15

**Authors:** Jinook Lee, Moon Gi Kang

**Affiliations:** School of Electrical and Electronic Engineering, Yonsei University, Seoul 03722, Republic of Korea; jin727@yonsei.ac.kr

**Keywords:** model-based image signal processor (ISP), differentiable ISP, cross-camera adaptation, single-pair calibration, test-time optimization, RAW-to-sRGB mapping, camera JPEG, color correction

## Abstract

Every camera carries an image signal processor (ISP) that converts raw sensor data (RAW) into a viewable sRGB image; each body needs its own tuning. An ISP trained on one body degrades on another, and adapting a learned ISP measures how far it falls short, not why. We adapt a hybrid ISP instead: a deep-learning controller predicts the seventy named physical coefficients of a differentiable model-based pipeline, so what the pipeline can express is known before measurement. One calibration pair (RAW, sRGB) refits those coefficients to an unseen camera; the ISP then deploys alone, with no added weights and no per-image network pass. The one color transform the pipeline cannot express is predicted before measurement, and so is where its error appears. The five unseen bodies are four DSLRs and one smartphone. Refitting all coefficients to each test image’s own ground truth sets the best the pipeline can reach, and one pair recovers most of it. The predicted deficit appears on one body in the predicted band; the measurement decides which body. A closed-form color stage fitted from the same pair recovers +3.28 dB of peak signal-to-noise ratio (PSNR) there. A gate that reads only calibration data applies it to that body alone. The deployed ISP runs at 341× fewer multiply–accumulates per pixel than a weight-space reference retrained by us. The corrected fit still trails that reference; the remainder is unattributed.

## 1. Introduction

Every camera contains an image signal processor (ISP) that converts raw sensor data into a viewable sRGB image. It consists of a fixed set of modules: white balance, demosaicing, a color-correction matrix, and a tone curve. Each manufacturer tunes those same modules a little differently for every body it sells. Most recent research replaces that pipeline, training one neural network that renders end-to-end on paired RAW–sRGB data [1,2]. We keep the module pipeline and put a network in front of it, predicting the modules’ coefficients rather than replacing the modules. The question is then what it takes to retune those coefficients for a camera the network has never seen.

### 1.1. The Cross-Camera Gap and Its Missing Cause

An ISP is body-specific. Two sensors under one illuminant render the same scene to visibly different sRGB images, so an ISP trained on one body degrades on another. A learned ISP regresses the whole pipeline from RAW–sRGB pairs [1,2], and the convention is to retrain it per body on a new paired dataset. The cheapest alternative is calibration: adapt the existing ISP from a single captured (RAW, sRGB) pair.

Existing ISP-adaptation methods—parametric fine-tuning of a physical pipeline [2], augmentation consistency on one test input [3], entropy-driven test-time adaptation [4]—report the score reached on the new camera. They do not report the best score those parameters could have reached, so a gap remains between the two. That gap cannot be interpreted. A few decibels may mean too few parameters, too little optimization, too little information in one pair, or a real gap in a per-image network. An end-to-end network is not divided into functional modules, so no score can say which part fell short.

### 1.2. What We Derive, Measure, and Correct

Our position is that the parameters moved at test time should be the ones a camera engineer already names. Our aim is a cross-camera adaptation whose reachable set of transforms is known before any image is scored. Naming the operators makes that set derivable, and a gap then names the operator it belongs to.

**Figure 1:**
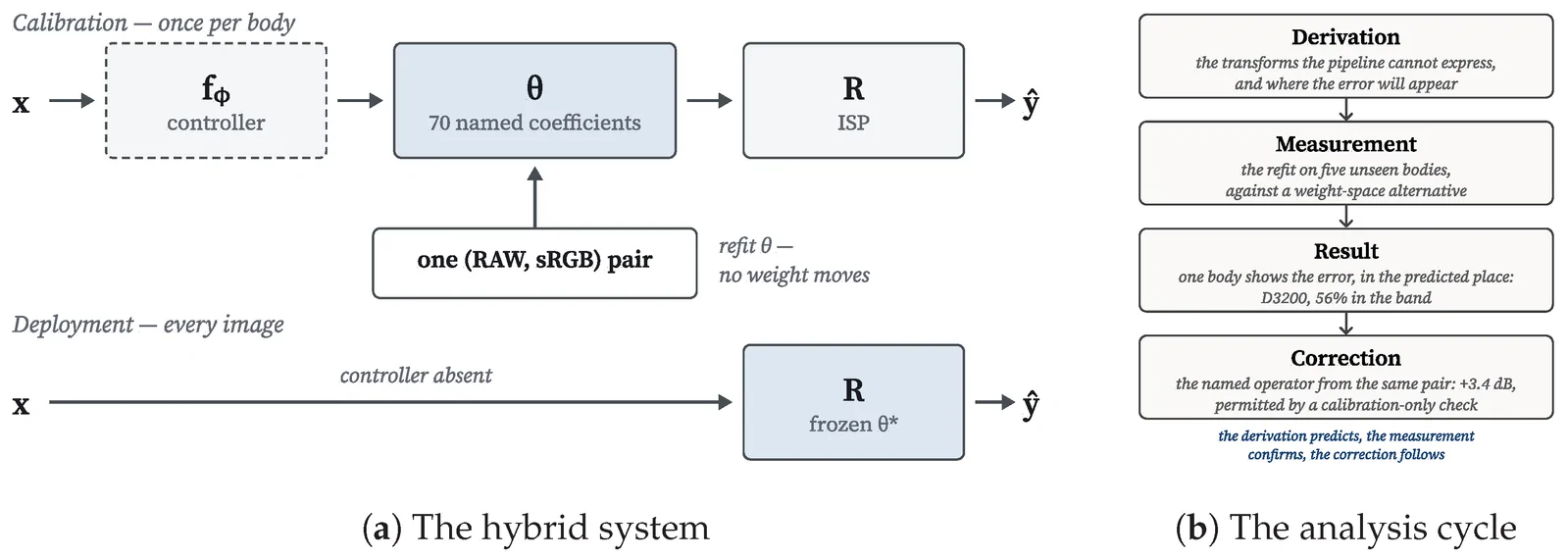
The system and the argument. (**a**) A deep-learning controller predicts the seventy named coefficients of a model-based ISP; calibration refits them and deploys the ISP alone. (**b**) The analysis cycle the naming enables: derivation, measurement, result, correction.

*Adapt* here means:

(1) $$\hat{y}\;=\;R\;\left(x,b;\;\theta ,\pi \right),\;\theta \;=\;f_{\phi }\left(x,m\right),$$

where a differentiable model-based ISP *R* renders packed RAW *x*, with Bayer pattern *b*, as $\hat{y}$. Inside *R*, the modules and their order π remain the same from camera to camera; only the seventy physical coefficients θ change. The starting point for θ comes from a controller $f_{\phi }$, a network trained once on one source body (Section 3.1). Calibration then refits θ alone on that single pair; no network weight is touched. At deployment the controller is removed, and the ISP runs alone (Figure 1a).

***Derivation.*** Because every module has a known algebraic form, the transforms this pipeline cannot express follow before any image is seen (Section 3.1.1), as does where the error will appear on a camera that needs one.

***Measurement.*** On five unseen cameras, we refit the pipeline to each test image’s own ground truth, to find the best it can reach. We compare that with an alternative that learns weights (Section 3.3).

***Result.*** Only one of the five cameras shows the error and in the predicted place (Section 4.2.1).

***Correction.*** Since the missing piece is named, we add that piece alone, fitted from the same calibration pair. A check that uses only calibration data permits the stage on that camera alone (Section 4.2.3).

### 1.3. Contributions

We characterize single-pair cross-camera adaptation of a hybrid ISP against camera-JPEG targets. The five unseen bodies are four Nikon DSLRs (D40, D90, D750, D3200; Nikon Corporation, Tokyo, Japan) and a Samsung S7 (Samsung Electronics, Suwon, Republic of Korea) mobile sensor. The pipeline is trained on the Nikon D7000 alone. Our contributions are as follows:

- *A single (RAW, sRGB) pair is enough to adapt the ISP to a camera it has never seen. Only seventy named coefficients change, and the camera ships with the ISP alone.* Three pairs recover +1.99 to +9.12 dB over the zero-shot render on all five bodies. With the bilateral denoiser the deployed ISP runs at 341× fewer multiply–accumulates per pixel than the weight-space reference (Section 4).
- *Because every module has a known formula, we can say before testing which operation the pipeline lacks and how its error would look. On five cameras, that error appeared on exactly one, and where predicted.* What survives the correction we report unattributed (Section 4.2.1 and Section 5.6).
- *Adding just that missing operation, fitted from the same calibration pair, recovers the predicted loss. A check on the calibration data alone decides which camera needs it.* The stage recovers +3.28 ± 0.46 dB on the D3200. The four controls remain within their own trial scatter, and the gate withholds the stage from all four (Section 4.2.3).

The remainder of the paper proceeds as follows. Section 2 sorts prior work by whether its expressible set is knowable. Section 3 specifies the system, derives what its composition can express, and sets the decision rule. Section 4 raises the calibration budget step by step, from the refit ceiling to one pair to more pairs. Section 5 states what the margins measure, which body completes the sequence, what the controller contributes, and where the claims stop.

## 2. Related Work

What decides a class’s relevance here is whether the set of RAW-to-sRGB maps it can express is knowable before measurement. Named algebraic form lets a gap be interpreted against an operator; rendering absorbed into weights leaves it as a score to report. We omit inverse-ISP work [5,6]. We also omit ISP retuning and resequencing for a machine consumer [7,8,9,10,11,12], whose objective is a task metric rather than the camera’s own JPEG rendering.

### 2.1. ISPs with Explicit Modules: The Expressible Set Is Derivable

The first class keeps the classical module chain explicit, so its expressible set is derivable in principle. A software platform reproduces camera pipelines stage by stage [13], and an inverted pipeline synthesizes RAW for denoiser training [14]. A differentiable pipeline can also sit inside a denoising objective [15]. All three use the chain as a tool. Our ISP fits the coefficients of the same chain to a calibration pair. Colorimetric characterization fits one such operator on its own. A brightness-preserving 3 × 3 least-squares matrix on linear RAW [16] imposes the constraint our row normalization does. Kernel partial least squares [17] or Gaussian-process regression [18] fit a free-form map instead. Those fits stop at a device-independent space read off a chart; the rendering recovered here carries the body’s own tone and saturation from natural scenes.

### 2.2. End-to-End Learned ISPs: The Expressible Set Is Not Knowable

End-to-end learned ISPs regress the camera rendering directly from paired data [19,20,21,22], commonly on a general-purpose restoration backbone [1]. ParamISP [2] adds the camera’s capture metadata as a conditioning input. Unlike the first class (Section 2.1), network weights absorb the rendering. A new body needs new paired data, and when the rendering is wrong, there is no white-balance gain, tone curve or color matrix to inspect. Nothing states which renderings the weights can and cannot produce. The gap therefore cannot be interpreted, however precisely it is measured, a limit every study adapting purely in weight space inherits. The class carries no single-pair protocol of its own. Published cross-camera figures attach to different sensor pairs, different targets and full paired training sets on the target camera. To our knowledge, no published ISP, end-to-end or modular, adapts to an unseen body from at most five calibration pairs against its own camera JPEG.

### 2.3. Test-Time Adaptation: Parameters Without a Named Set

Test-time adaptation moves parameters whose expressible class is again not knowable. Entropy minimization [4] and augmentation consistency [3] adapt a trained model on the test distribution. Both criteria target classification and do not tie to a camera’s rendering, so neither is expected to identify the target here. Supervised test-time updates are the relevant comparison; low-rank weight adaptation [23] is the form we compare against (Section 3.3).

### 2.4. Domain Adaptation, Color Constancy, and Neural ISP Simulation: The Same Side of the Axis

Three neighboring lines sit on the same side of the axis above. Unsupervised domain adaptation moves weights toward an unlabeled target domain by aligning feature distributions [24,25]. The expressible set is again not knowable, and we use the supervision of one calibration pair rather than a pool of unlabeled images. Learned color constancy trains one camera-agnostic network to estimate the illuminant [26], or calibrates itself on an unseen camera from unlabeled images at test time [27]. Our white-balance gains are named coefficients inside the pipeline, refitted per body from one pair. Neural ISP simulation imitates a camera’s rendering and capture settings with weights [14,22,28], and a simulator does not state what it cannot express. All three leave the expressible set unknown before any measurement.

## 3. Proposed Method

Section 3.1 describes the modules of our ISP and their seventy coefficients, then writes in one line what those modules do to a color. It also describes the controller that predicts the coefficients. Section 3.2 covers everything fitted from one calibration pair. Those are the seventy coefficients and, from the same pair, the modules themselves: whether to add a color stage and which denoiser to use. What that composition excludes is derived at the end of Section 3.1.1; how the exclusion is tested is the subject of Section 3.3.

### 3.1. Hybrid System: Deep-Learning Controller, Model-Based ISP

Write $x\in R^{H\times W\times 4}$ for a packed Bayer RAW image and $y\in {\left[0,1\right]}^{2H\times 2W\times 3}$ for the sRGB image the camera itself produced from it. The Bayer pattern is *b*, and *m* is a 20-dimensional vector of file metadata. Its twenty values are the camera-to-sRGB matrix, three as-shot gains, log ISO, exposure time, f-number and focal length, and per-channel black and white levels. In Equation (1), θ is a 70-dimensional physical parameter vector, interpretable stage by stage (Table 1). The controller $f_{\phi }$ predicts it from the RAW input, *b* and *m*.

#### 3.1.1. Differentiable Classical Pipeline

**Figure 2:**
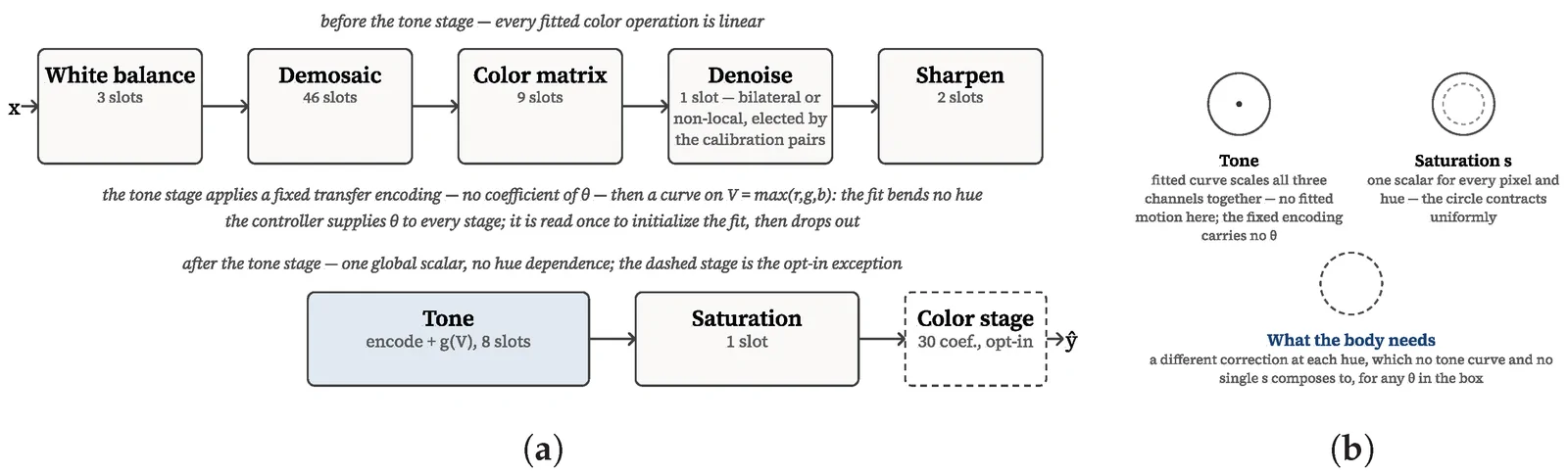
The ISP. (**a**) The chain in execution order and its slots (Table 1); the dashed stage is the optional module of Section 3.2. (**b**) The hue–saturation plane at fixed luma.

*R* is a conventional module chain, differentiable in every parameter; Figure 2 lays out the execution order, while Table 1 shows the slots and ranges. Four operators touch color: white balance, the color matrix, the tone curve and the saturation gain.

***White balance and color correction.*** Two pre-tone operators act on color: per-channel gains, the two green sites sharing one gain, and a row-normalized 3 × 3 matrix *M* with no bias term. Both are linear in the pixel, their coefficients shared by every pixel, and neither depends on hue before acting.

***Demosaicking.*** A gradient-based threshold-free kernel [29] interpolates the mosaic to a full RGB image along inter-channel color differences. The forty-six scalars of Table 1 scale its taps near identity, and observed samples pass through unchanged. At an edge the output at a pixel is therefore interpolated from neighboring samples, not a function of that pixel’s color alone.

***Denoising and sharpening.*** Two spatial operators follow the color matrix, and neither mixes channels. The denoising slot holds one of two parameter-free operators under the same strength coefficient. By default, it is a bilateral filter [30]; where the calibration pairs elect it, it is non-local means [31] (Section 3.2). An unsharp mask follows.

***Tone.*** The stage first applies the transfer encoding of the body’s tagged output color space. This is sRGB’s standard piecewise curve, or Adobe RGB’s pure power law where the file’s ColorSpace tag says so (sRGB where the tag is absent). That encoding is channel-identical, fixed, and carries no coefficient of θ. In the encoded coordinates, a monotone piecewise-linear curve *g* then applies to V=max(r,g,b) and rescales the triple by the ratio,

(2) $$T\left(c\right)\;=\;c\cdot \frac{g(V)}{V},\;V=\max\left(r,g,b\right),\;c\in \left\{r,g,b\right\},$$

where *g* is a positive-weight (softplus) combination of the eight slots with g(0)=0 and g(1)=1. Every channel is multiplied by the same positive scalar g(V)/V, so the RGB direction, hue and saturation are unchanged. The curve takes *V* to g(V) and nothing else, at every hue alike.

***Saturation.*** One operator closes the chain: a saturation gain $c\mapsto \bar{c}+\left(c-\bar{c}\right)\;s$ with $\bar{c}$ the BT.709 luminance [32]. The gain moves colors along rays through the neutral axis at a single rate *s* shared by every pixel and hue. It is the only operation after the tone stage.

***Composition.*** Call $c_{p}$ the color of one pixel as it arrives at the tone stage. Every stage before the tone curve, the three spatial operators included, is already inside $c_{p}$. From the tone stage on, the chain does one thing to that color:

(3) $${\hat{y}}_{p}\;=\;\left(\;{\mathrm{sat}}_{s}\circ T_{g}\;\right)\;\left(c_{p}\right).$$

The tone curve $T_{g}$ and the saturation scalar ${\mathrm{sat}}_{s}$ are all that acts on $c_{p}$. Across the whole chain, θ changes a color in four ways only: white-balance gains, color matrix, tone curve and saturation scalar.

***What the pipeline order excludes and predicts.*** The tone curve changes only the brightness V=max(r,g,b) and leaves hue and saturation as they are (Equation (2)). After it, the only operation that touches color is the saturation scalar ${\mathrm{sat}}_{s}$ (Equation (3)). A color transform that acts differently on different hues after the tone stage is therefore outside the class for every θ in the box (Figure 2b). That is a property of the function class, settled before any camera is measured. The spatial stages do not change this. Demosaicking interpolates from neighboring samples, so the output at a pixel is not one 3 × 3 matrix applied to that pixel’s color. It runs before the tone stage and is inside $c_{p}$. Denoising and sharpening also run before the tone stage and mix no channels, so no spatial operator acts after it. Treating the pre-tone stages as a per-pixel map is exact only where color is locally uniform. The demosaicking residual at edges lands in high-frequency luma, which Section 4.2.1 measures on its own. The prediction rests on one assumption: natural-scene chroma concentrates at low spatial frequency, and the error of a per-pixel color function inherits that spectrum. On a camera that needs the missing transform, the error must therefore appear in low-frequency chroma (Section 4.2.1). The exclusion is inexact in one place. A channel that reaches the gamut boundary is clipped, and that clip acts differently on different hues. It carries no coefficient of θ and does not occur in the gamut interior.

#### 3.1.2. Controller and Training

The controller is a convolutional encoder that maps the RAW input to θ. It is trained once on a single source body, the Nikon D7000, against that camera’s own JPEGs; no evaluation-body image is seen at training time. It serves two roles: the zero-shot prediction reported as the no-calibration result, and the initialization of the fit below. The metadata vector *m* only enters the former; the initialization substitutes a neutral constant for *m*, and no ISP stage reads *m*. What that initialization is worth is measured in Section 5.4.

***Architecture.*** The encoder follows NAFNet [1]. A 3 × 3 convolution lifts the four packed Bayer channels to 32 features. Four stages of 2, 2, 2 and 1 NAF blocks follow, at widths 32, 64, 128 and 256. A stride-2 convolution sits between stages, so the final feature map is one eighth of the packed size. A two-layer MLP embeds *m*, and the embedding modulates the stage features by feature-wise affine transforms. A slot-query attention pool then extracts one 256-dimensional descriptor per parameter slot. A per-slot MLP with a sigmoid output places each coefficient in the normalized box [0,1] of Table 1.

***Training.*** The training set is the 5200-image training split of RAISE D7000; 604 images are held out for validation by a seeded random permutation. Each step draws a random 512 × 512 Bayer patch with a Bayer-aware horizontal flip, the only augmentation. The loss is the L1 distance to the camera JPEG. Adam runs at learning rate 5 × 10^−4^ with weight decay 10^−4^, a 500-iteration linear warm-up, then cosine decay to one percent of the peak. The batch is four images per GPU on two GPUs, and gradients are clipped at 1.0. Training runs for 100 epochs, and the checkpoint with the highest validation peak signal-to-noise ratio (PSNR) is kept. There is no cross-validation, and no evaluation body enters model selection.

### 3.2. Single-Pair Calibration: Fitting the Coefficients and the Modules

***Fitting the coefficients.*** The composition fixes what any value of θ can express; what remains is to choose that value for an unseen body. Adaptation fits Equation (4) on *k* calibration pairs ${\left\{\left(x_{i},y_{i}\right)\right\}}_{i=1}^{k}$ alone, with k=1 unless stated otherwise. This fit is the test-time optimization of the title,

(4) $$\theta^{★}\;=\;\arg\min_{\theta \in [\ell ,u]}\;\frac{1}{k}\sum_{i=1}^{k}{∥R\left(x_{i};\theta \right)-y_{i}∥}_{1},$$

by Adam for 200 steps at learning rate 5 × 10^−2^. The optimization runs in box-normalized coordinates, each parameter mapped affinely from its range in Table 1 onto [0,1]. Gradient norms are clipped at 1.0, and the returned parameters are clamped to the box. Each step evaluates the objective on a fresh random 512 × 512 crop of each pair, drawn from a generator seeded with the trial index. The fit starts from $\theta_{0}=f_{\phi }\left(x_{1}\right)$ and keeps the iterate with the lowest calibration loss. We call the resulting $\theta^{★}$ *the physical fit* throughout.

***Fitting the modules: the color stage.*** One standard operator supplies what the composition lacks, appended to the rendered output where a body needs it (Section 3.1.1). It is the degree-two polynomial color transform of classical colorimetric characterization [33,34],

(5) $$\tilde{y}\;=\;A\;p\left(\hat{y}\right),\;p\left(c\right)={\left[\;1,\;r,\;g,\;b,\;rg,\;rb,\;gb,\;r^{2},\;g^{2},\;b^{2}\;\right]}^{\;⊤},\;A\in R^{3\times 10}.$$

Because it acts after the tone stage, it lies outside the class in every form. Camera processing carries exactly such a stage. The DNG specification holds hue- and saturation-dependent profile tables [35], and the platform of Karaimer and Brown applies a photo-finishing color manipulation [13]. A ridge fit shrunk toward the identity estimates its 30 coefficients in closed form from the *same* calibration pairs. No test ground truth and no retraining enter this fit. The ridge weight λ is fixed at 10^−4^ of the mean diagonal of the monomial Gram matrix. The same value therefore acts at the same scale on every body. The stage is fitted from the pairs, but it is off by default. Whether it is adopted is decided by a *calibration-only gate* that runs leave-one-out on the same pairs. For each held-out pair, the gate fits *A* on the remaining pairs and measures the change in PSNR on that pair,

(6) $$\mathrm{LOO}\;=\;\frac{1}{k}\sum_{i=1}^{k}\left[\mathrm{PSNR}\left(A_{-i}\;p\left({\hat{y}}_{i}\right),\;y_{i}\right)-\mathrm{PSNR}\left({\hat{y}}_{i},\;y_{i}\right)\right],$$

and applies the module only when LOO>0; the rule never inspects a test image. Holding out one pair means Equation (6) needs k≥2, though the stage itself fits from a single pair. The decision is the mean over five calibration trials per body, with the number of individually positive trials reported alongside. The criterion is PSNR-only; whether its decisions agree with the test-side outcome is measured in Section 4.2.3.

***Fitting the modules: the denoiser.*** The same procedure chooses the operator in the denoising slot. Both candidates are parameter-free and read the same strength coefficient, so one fitted θ renders under either. The pipeline starts with the bilateral filter, the lighter of the two. Non-local means costs an order of magnitude more work in the slot and must justify that cost. At calibration, the pairs are refit under each operator, and the one with the higher calibration PSNR is kept. It is an argmax on the calibration data, one decision per body; no test image enters. Its cost is a second calibration fit on the heavier candidate. Section 4.2.2 reports the cost of both configurations. This gate and Equation (6) are the pipeline’s only structural choices, and both read calibration pairs alone.

### 3.3. Diagnostic Design

**Figure 3:**
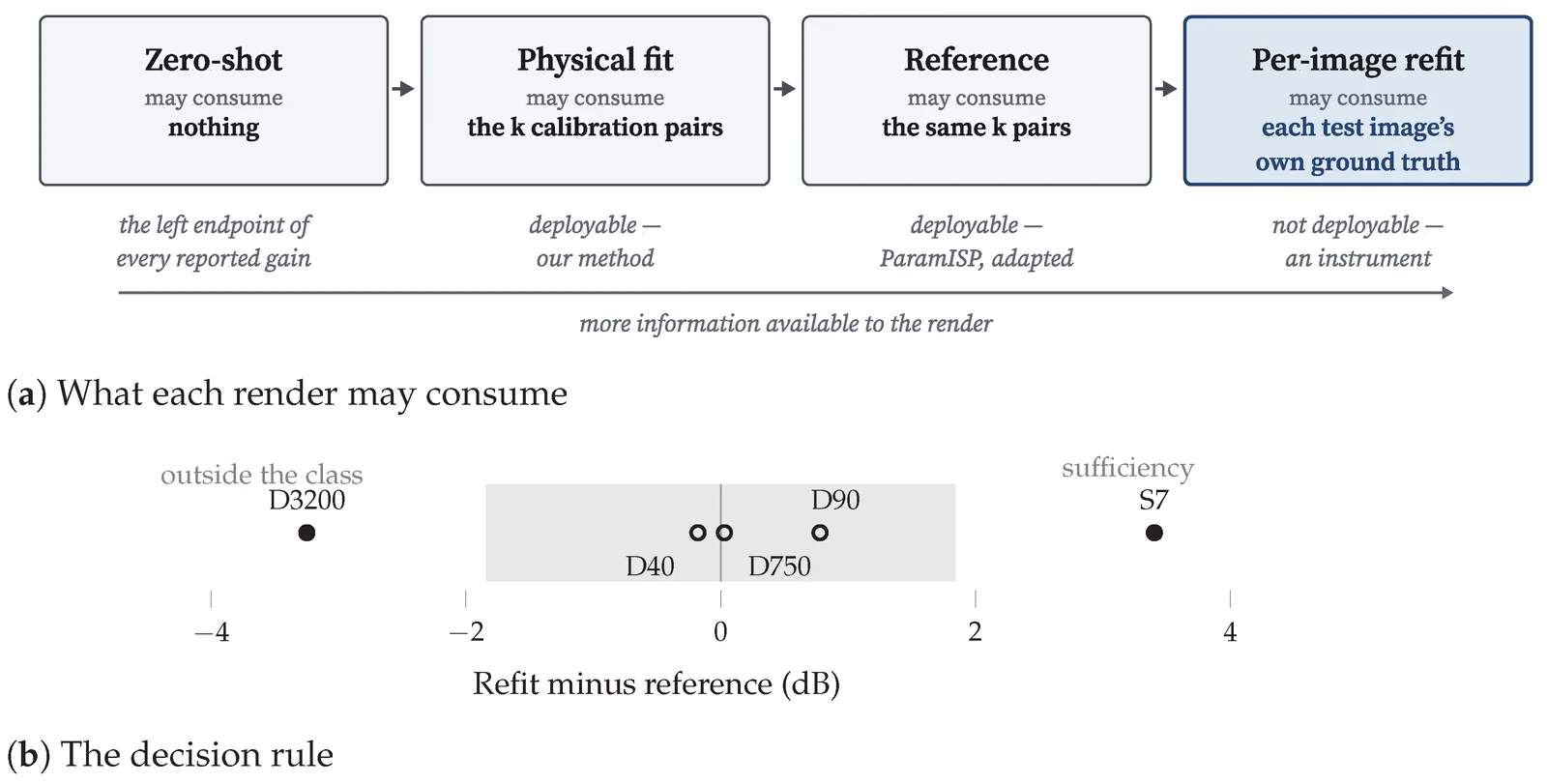
(**a**) Four renders of one body, by what each may consume; the refit consumes each test image’s own ground truth. (**b**) The decision rule: inside the shaded band, the comparison’s largest movement of ±1.84 dB, a margin decides nothing.

***Three reference points.*** Three reference points separate what the class can reach from what one pair recovers, and they differ in what each may see (Figure 3a). The zero-shot render sees nothing and is the left end of every gain. The weight-space reference sees the calibration pairs only, so it can be deployed. The per-image refit sees each test image’s own ground truth, which shows how far the class reaches. The refit solves the objective of Equation (4) per test image against that image’s own ground truth:

(7) $$\theta_{j}^{o}\;=\;\arg\min_{\theta \in [\ell ,u]}{∥R\left(x_{j};\theta \right)-y_{j}∥}_{1},\;\mathrm{evaluated}\;\mathrm{as}\;R\;\left(x_{j};\theta_{j}^{o}\right).$$

It uses the same ISP, gate-elected denoising operator, optimizer and initialization rule, at its own rate and budget (learning rate 10^−2^, 800 steps). It is a local optimum of a mean-absolute-error objective on the sRGB output; what it reports is a lower bound on what the class reaches. The weight-space reference is the published ParamISP architecture [2], retrained by us on the controller’s source body. It is adapted on the same calibration pairs through a rank-one update [23]. It receives the same data and the same adaptation as the physical fit (Section 4.1.2).

Two terms are used from here on:

**Margin**  The signed PSNR or SSIM difference by which the per-image refit leads or trails the weight-space reference on one body.
**Resolution**  The largest movement the comparison itself makes with the body fixed, here ±1.84 dB; no smaller margin decides anything.

***Decision rule.*** A margin becomes a finding only past the resolution. The resolution is built from the refit’s sensitivity to its configuration, one decade of the reference’s fitting rate, and the across-trial spread (Section 4.2.1 and Section 5.1). It exists in PSNR alone; SSIM margins are reported beside it and decide nothing. Past it, a shortfall places what the body needs outside the class, jointly with the algebra’s naming (Section 3.1.1) and the closed-form control (Section 4.2.1). A surplus shows the class contains settings of that quality. Inside the resolution nothing is decided.

## 4. Experiments

Section 4.1 fixes the experimental conditions: the five cameras and each camera’s own JPEG as the target (Section 4.1.1). It also counts uncertainty per calibration trial, states the cost accounting, and defines the three network adaptations we compare against. Section 4.2 raises the calibration budget one step at a time. More specifically, Section 4.2.1 refits all seventy parameters to each test image’s ground truth and measures how far the pipeline reaches and why it stops there. Section 4.2.2 measures how much of that ceiling one pair recovers and at what cost. Section 4.2.3 measures what further pairs add. Section 4.3 shows the same results as rendered images.

### 4.1. Setup

#### 4.1.1. Cameras, Trials, and Metrics

***Cameras.*** Every evaluation body is unseen during training, and every target is the JPEG that body itself produced for the exposure. The task is therefore to reproduce a specific camera’s rendering. Table 2 lists the bodies, their sources, sensors, capture notes and image counts. The five bodies are single-frame Bayer pipelines: four Nikon DSLRs of the training (among public DSLR RAW datasets, the Nikon collections are the ones we found that ship each frame’s own full-size JPEG intact, without re-encoding or resizing) and one 2016 smartphone. The five evaluation sets come from four datasets and differ in scene content. The D750 set is night scenes, and the D3200 set was shot under one fixed manual white balance (Table 2). On the Samsung S7 the decoder returns an all-zero color matrix for every file, so we rebuild it from the DNG ColorMatrix2 tag. Only the controller’s zero-shot metadata and the reference read it. The physical fit, the refit and the color stage read no metadata.

***Trials.*** A *trial* is one random choice of *k* calibration pairs followed by the fit; each body has five trials. On the D40 at k=1, two of them draw the same pair. A calibration pair is drawn from the same body without using its ground truth for selection. Each trial draws a random pool of ten images from the body, seeded with the trial index. A greedy furthest-point rule orders that pool by nine per-image statistics: three channel means, brightness spread, dynamic range and four log-EXIF values. The *k* pairs are the first *k* of that order, so the one-pair set lies inside the three-pair set. No evaluation image enters the choice. Every five-body result, the gate and the initialization study use the same five trials per camera, and uncertainty is counted per trial. Held-out counts shrink with *k*: 71–75 (D40), 215–219 (S7), 495–500 (D90) and 496–500 (D750, D3200).

***Metrics.*** The headline metrics are full-resolution PSNR and structural similarity (SSIM) [40] against the camera JPEG under a sliding-window evaluation, and both are reported wherever they disagree. Where SSIM is reported under a center crop, the crop ratio is 0.8.

The learned perceptual image patch similarity (LPIPS) [41] is reported beside them.

#### 4.1.2. Compared Methods

The comparison is against ParamISP [2], the state-of-the-art learned ISP conditioned on camera parameters. We trained it with the authors’ released code, from scratch, on the source body’s own camera JPEGs (Nikon D7000), with no published weights entering (Section 2.2). The base has 709,885 parameters. Paired margins (Section 4.2) require the reference to share the physical fit’s source body, data and pairs; published ParamISP weights and results come from other cameras. A learned ISP becomes a reference on an unseen body only through its own adaptation. The base therefore receives the same k=3 pairs the physical fit uses, and three adaptations. Each narrows, step by step, how much of the network the optimizer may move. *ParamISP-FT* moves every weight (rate 3 × 10^−4^). *ParamISP-tail* moves the decoder block alone, 34,627 weights (rate 10^−3^). *ParamISP-LoRA* moves rank-one adapters [23] on the frozen base’s 57 convolutions, 54 local and 3 tone, for 27,481 trainable weights (rate 3 × 10^−3^). The rank-one variant is the weight-space reference of Section 3.3; it receives the same data and the same adaptation as the physical fit.

The rest is shared: Adam for 200 steps, gradient norms clipped at 1.0, and one fresh random 512 × 512 crop of each calibration image per step. The objective is Charbonnier, where the physical fit’s is the $L_{1}$ of Equation (4), and the step-200 iterate is deployed.

***Choosing the reference among four mechanisms.*** Table 3 places the four mechanisms on one D90 trial of three pairs. Inside one function class, how much of the pipeline the fit may change is worth 2.34 dB. The step to the seventy physical parameters, a change of function class, is a single-trial point comparison. From the gate-elected row, it is 2.67 dB to the nearest weight-space mechanism and 5.01 dB to the full fine-tune’s median. Both exceed the 2.34 dB spread inside weight space. Inside weight space, the spread does not follow parameter counts: the tail decoder moves 34,627 weights and scores below the rank-one update’s 27,481. SSIM ranks the four identically.

#### 4.1.3. Cost Accounting and Measurement Conditions

Every cost in Table 3 is measured under one convention, the same for the ISP and for the weight-space mechanisms. The counted path is the one executed after calibration. For the hybrid ISP, that path excludes the controller, which runs once at calibration, and includes every stage of the configuration the gate elected. Table 3 reports both configurations. The color module counts only where its gate fires. Multiply–accumulates are counted over the operations each forward pass dispatches on one 512 × 512-output tile. The count is one per input element consumed by a product–sum or reduction and one per output element of every elementwise multiplication, division or transcendental. Additions, comparisons, clamping, padding and data movement count zero, and the total is divided by the tile’s output sRGB pixels. Latency is the median wall-clock time over timed repeats after warm-up, for one full-resolution image on one GPU. Both systems use the same tiling, batch size and precision. Active parameters are those the deployed path evaluates. Calibration time is the median over five timed fits on freshly built models after a discarded warm-up, excluding data loading, model construction and checkpoint reading.

All timings run on one NVIDIA GeForce RTX 5090 (NVIDIA Corporation, Santa Clara, CA, USA) in fp32, with automatic mixed precision, TF32 and cuDNN autotuning off, under PyTorch 2.10.0 with CUDA 12.8. Inference is timed on a 6.016 MP frame (2000 × 3008 Bayer), with 10 warm-up and 50 timed repeats. Calibration is timed as 200 Adam steps on three fixed D90 pairs. The CPU columns repeat the same protocol on one core of an Intel Core i9-14900KF (Intel Corporation, Santa Clara, CA, USA), with PyTorch 2.10.0 limited to one thread. Inference uses 3 warm-up and 10 timed repeats; entries above one minute are single runs. Calibration is the median of three timed fits for the physical fit and a single fit for the network rows.

### 4.2. Quantitative Analysis

#### 4.2.1. What the Pipeline Can Reach

**Figure 4:**
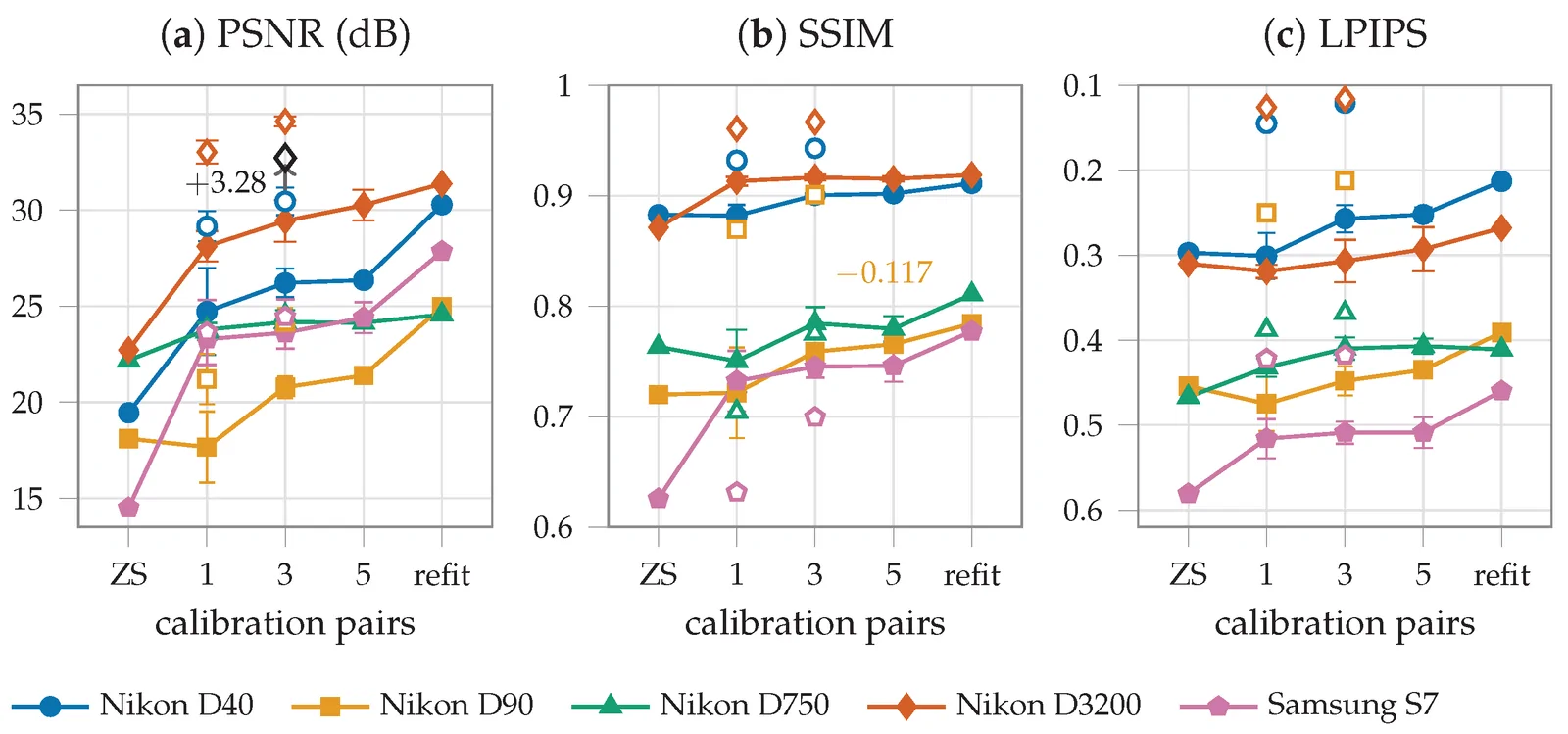
Fidelity against the calibration budget, per body. Filled markers: the physical fit, bars the across-trial standard deviation; open markers: the reference. The refit column consumes test ground truth. (**a**) PSNR; (**b**) SSIM; (**c**) LPIPS, lower is better.

***Against the network reference.*** The refit ceiling is read against the reference under the one-sided rule of Section 3.3. The resolution is how far either side moves under its own settings. Configuration selection (Section 3.2) shifts the refit’s per-body PSNR by 0.20–1.84 dB. One decade of the reference’s fitting rate is worth 1.0–1.7 dB, and its across-trial spread reaches 0.90 dB. Read against movements of that size, Table 4 returns three decisions. On the S7, the margin is +3.403 dB, nearly twice the largest movement, and SSIM agrees at +0.078. Settings of the seventy parameters that reach past the reference exist. On the D3200, the margin is −3.248 dB, well past that movement, and SSIM agrees in sign at −0.048. The refit alone cannot carry that exclusion, being a local optimum. The exclusion is the algebra’s, fixed for every θ in the box before any image was scored (Section 3.1.1), and this margin sizes it. The closed-form control of Section 4.2.1 rules the search alternative out. On the D40, D90 and D750 the PSNR margins of −0.179, +0.779 and +0.029 dB all fall inside those movements. Under SSIM, the reference leads on all but the D750 and S7, where the fit leads (Figure 4b). The D90’s paired SSIM runs against the fit by 0.117, with no measured band, since the resolution is PSNR’s alone (Section 3.3).

**Figure 5:**
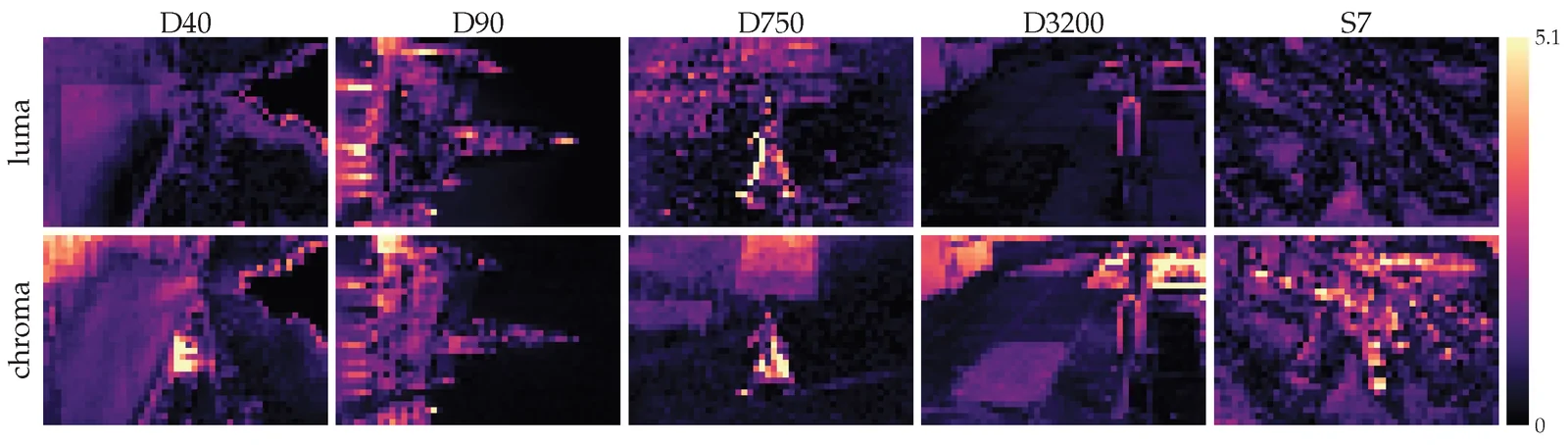
Pooled luma (**top**) and chroma (**bottom**) refit-error magnitude on the frames of Section 4.3, each body normalized by its own residual RMS; every panel consumes that frame’s ground truth.

***Where the remaining error concentrates.*** The derivation predicts where the error sits on every body. Where the transform that Section 3.1.1 places outside the class is missing, the error concentrates in low-frequency chroma. This test splits the refit’s *own* remaining error into luma and chroma across the DC, low, mid and high bands. It uses 30 images per body from one k=3 trial. Each body is scored by its share of residual power in chroma DC and low (Table 5). The prediction fixes the share, not the absolute power. The share is largest on the D3200, whose refit also stops furthest short of the reference (Section 4.2.1): 55.52 ± 4.87% against the D40’s runner-up 38.97 ± 6.17%. That gap of 16.55 points exceeds the combined 95% interval of 7.86. The remaining three, at 36.54, 20.37 and 7.50%, are separated neither from the runner-up nor from one another. In the luma high band, the shares reach 19.65%. The excess over the reference is decomposed in Section 5.2. The five bodies do not share one error profile. Because a share divides by each body’s own residual, the absolute chroma DC+low ordering differs. It runs D90, D750, S7, D3200, and D40, with the diagnosed body fourth (Section 5.3). The band separates where a residual sits; the identification and the correction belong to the D3200 alone. Figure 5 shows it: the D3200’s residual in the chroma row, the D750’s in the luma row.

***The missing operator raises that ceiling.*** Three things name the operator: the share above, the two measurements below, and the exclusion of Section 5.2. Without the operator, the refit stops at 31.371 dB. The class excludes post-tone hue-dependent transforms for every θ in the box. Refitting all seventy parameters per test image reaches no further on the D3200, a local optimum (Section 3.3). Supplying the operator raises what the refit reaches. The degree-three extension of Equation (5) has sixty coefficients. With it, the per-image refit reaches 34.13 dB on all 500 images, within the comparison’s resolution of the reference’s 34.62 (Table 6). This is an existence proof inside the post-tone family; the deployed stage itself is measured in Section 4.2.3. A simpler operator does not. On the same per-image refits (Table 6), three hue-independent per-channel offsets recover only +0.40 dB of the D3200’s shortfall, against +2.57 for the degree-two hue-dependent class. The degree-three extension above is the capacity control.

**Table 6:** PSNR added to the per-image refit by each post-tone operator, fitted in closed form on the same refits (test ground truth consumed); the last row is the reference at three pairs, which sees none. The deployable counterpart is Figure 6.

		D3200 (Diagnosed)		D40 (Control)	
Operator Supplied	Coef.	Refit	Δ	Refit	Δ
None	—	31.371	—	30.285	—
Per-channel offsets, hue-independent	3	31.769	+0.40	30.452	+0.17
Degree-two color transform, hue-dependent	30	33.940	+2.57	31.684	+1.40
Reference, k=3 (no test ground truth)	—	34.62	—	30.46	—

#### 4.2.2. What One Pair Recovers

***How much one pair recovers of that ceiling.*** A single calibration pair recovers +1.60 to +8.79 dB on four of the five unseen bodies (Table 4). On each of the four, that is most of the headroom below the refit ceiling (Figure 4). On the D90, the one-pair mean falls 0.44 dB under the zero-shot render. The zero-shot level itself spans 8.23 dB: the body matters more than the number of pairs. Against the reference fitted from the same pair, the fit trails by 4.44 dB on the D40, 3.54 on the D90 and 4.90 on the D3200. On the D750 and the S7, it sits within 0.36 dB of that reference (Table 4). Neither the DSLR-versus-mobile split nor the zero-shot level predicts that spread. The D750 and D3200 start within 0.54 dB of each other yet recover +1.60 against +5.39 dB at one pair. Two of seventy-four trials score below their own uncalibrated render, both single-pair trials on the D90.

**Figure 6:**
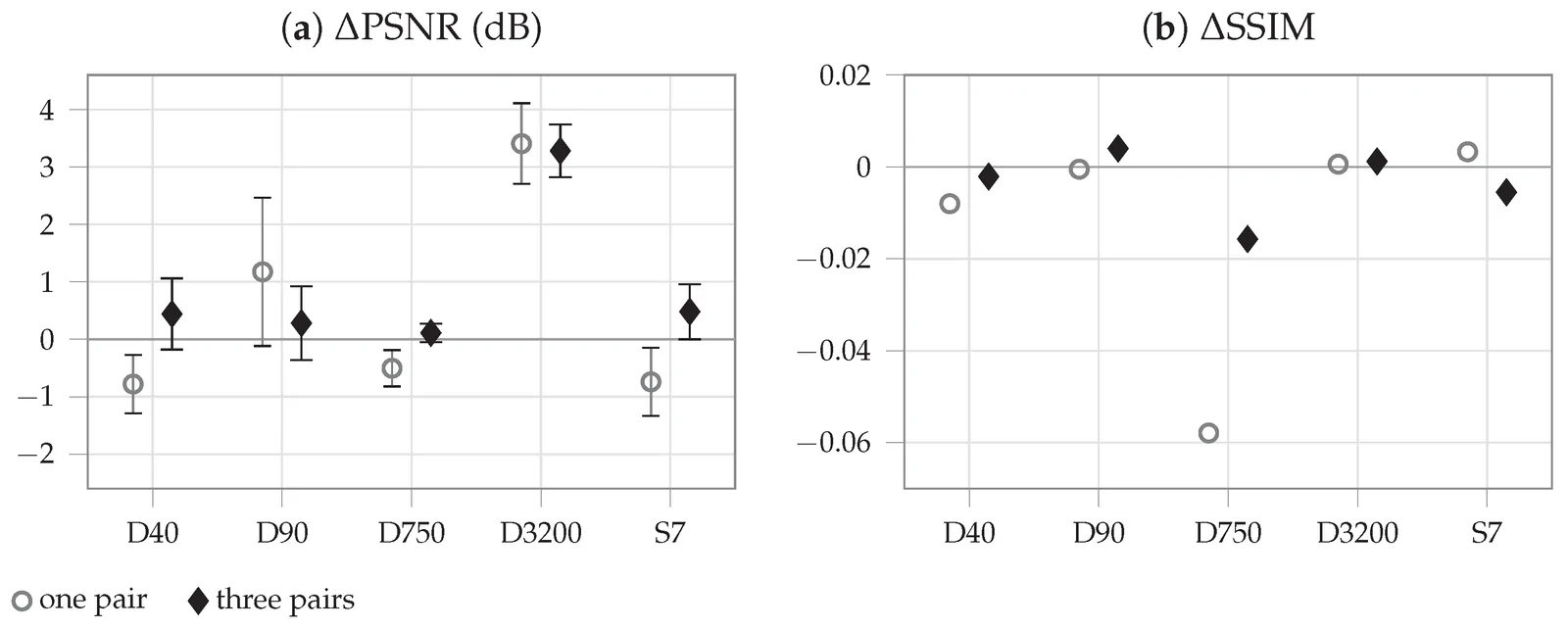
The color stage estimated from the calibration pairs alone, applied on top of the fit at one and three pairs; no test ground truth, mean over five trials, bars the standard deviation. (**a**) PSNR change; (**b**) SSIM change (center crop).

***One pair also supplies the missing operator.*** The same single pair also estimates the operator Section 4.2.1 named. The closed-form estimate of Equation (5) runs on that pair with the class and ridge weight fixed, the same as at three pairs (Section 4.2.3). Nothing is selected, and no test image enters. On the D3200, it adds +3.41 dB in PSNR, and SSIM agrees in sign at +0.0006 (Figure 6). There is no decision at this budget, since the leave-one-out of Equation (6) has nothing to hold out at k=1. The D3200’s spread across trials is 0.702 dB at the fit and 0.666 after the stage, with every trial gaining at least +2.75 dB. On the four controls, the same pair does not. Three move *down*, each clear of its own trial-to-trial scatter; the one nominal gain, the D90’s +1.17, falls inside its 1.29 spread.

***What it costs.*** Once calibration freezes $\theta^{★}$, the deployed path is the ISP alone; the controller’s 1,157,984 weights are read once, for the starting point. The three network mechanisms deploy their whole base regardless of what they adapt, and the rank-one update deploys more than the base (737,366 resident). The rank-one update moves twenty-six times fewer parameters than the full fine-tune yet finishes only 0.8 percent sooner. The tail decoder, moving more, is the quickest of the three. The physical fit’s gradient passes through no network. It therefore calibrates 5.5× faster than the rank-one update with the bilateral denoiser and 2.8× where the gate elects non-local means. That body also pays the gate’s second fit once. Which row applies is the gate’s decision. The bilateral row applies where the calibration pairs keep the bilateral slot—two of the five bodies. The non-local means row applies elsewhere and still uses 55.5× less arithmetic and 4.1× less latency than the reference. Its operation count is an upper estimate. The bilateral figures are 341× and 18.7× (Section 5.5). On one CPU thread, the latency margins are 5.3× for the non-local means row and 32.9× for the bilateral row. Calibration on the same thread is 4.1× and 17.3× faster than the reference (Table 3).

#### 4.2.3. What More Pairs Add

***What a second and third pair add.*** Three pairs recover between +1.99 and +9.12 dB on the five bodies, and the four DSLRs alone cover 4.78 dB of that 7.13 dB range. Per-trial SSIM and LPIPS agree with PSNR in direction on every body (Figure 4b,c). Adding pairs helps and stops helping early. From three to five pairs, the fit moves by at most +0.82 dB. Only the D90’s +0.61 dB stands above its own trial-to-trial scatter at both endpoints; no other body is resolved as still rising at k=5. What more pairs add more reliably than fidelity is predictability. The across-trial spread is smaller at k=5 than at k=1 on four bodies and unchanged on the D3200. Against the reference fitted from the same three pairs, the fit trails on every body (Table 4). The shortfall is 0.36 dB on the D750 and 0.83 on the S7. It is 3.39 on the D90 and 4.25 on the D40. On the D3200, it is 5.18 dB, and 1.90 once the operator is added (Section 4.2.3). That shortfall splits in two. One part is the class gap between the reference and the refit ceiling. The other is the cost of one θ per camera, between the ceiling and the fit (Section 5.3).

***Three pairs fit the operator more tightly.*** The closed-form estimate on the physical fit’s own k=3 pairs gives the stage’s 3 × 10 coefficient matrix, with no test ground truth and no retraining. It is applied to every evaluation image over five calibration trials (Figure 6). On the D3200, it is worth 3.28 ± 0.46 dB across trials on 498 images, both metrics agreeing, a narrower spread than the single pair’s 0.703. It costs one linear solution: the ridge fit runs no optimizer and takes no gradient step. Section 4.3 shows the corrected render on a crop, beside the reference.

**Figure 7:**
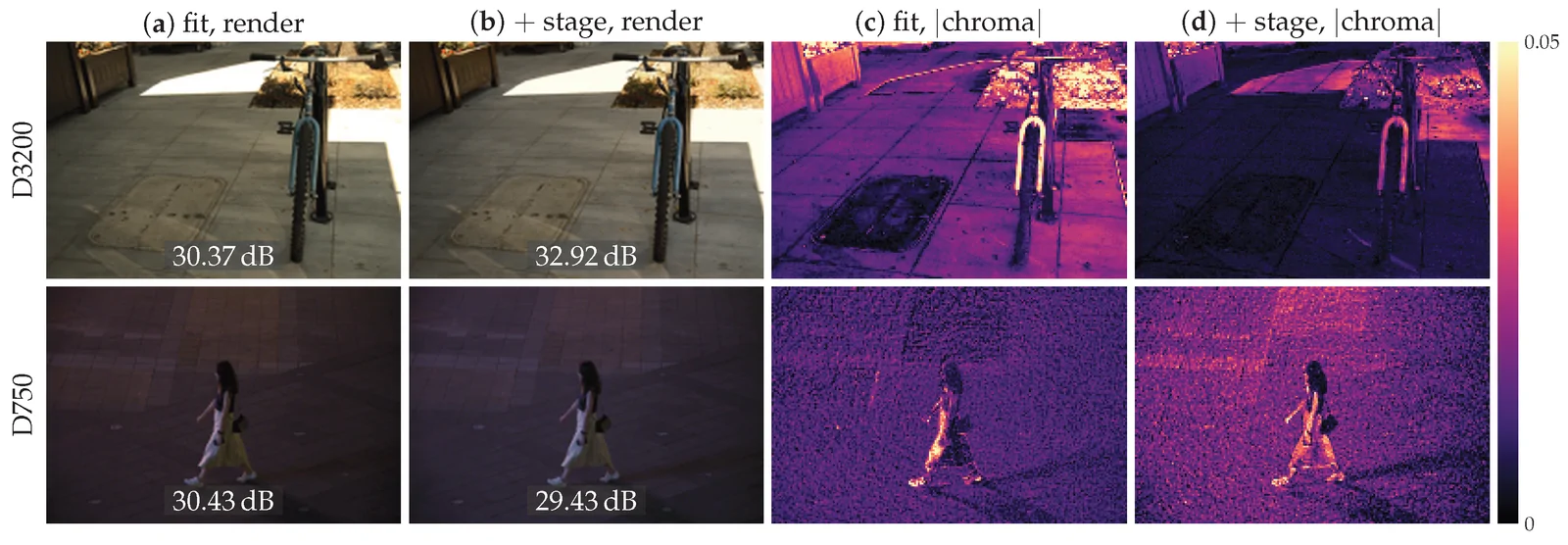
The k=3 fit before and after the calibration-only color stage on the frames of Section 4.3, with pooled chroma-error magnitude on one shared scale. No test ground truth enters any panel; overlays are each frame’s own PSNR.

***On the other four bodies, it adds nothing.*** On the four controls, the same estimate produces no gain the measurement resolves. The identical procedure on each body’s own pairs, applied unconditionally, moves them by at most +0.48 dB, each inside its own trial-to-trial scatter. It costs the D750 0.016 of SSIM. The D3200 meanwhile gains +3.28 dB with every trial positive (Figure 6): an identification rather than an increment. Where the pairs specify no body-constant correction, supplying the stage does not reproduce the gain. Figure 7 shows the identification as images. The same closed-form stage, fitted the same way, clears the D3200’s low-frequency chroma error and leaves the D750’s maps where they were.

**Figure 8:**
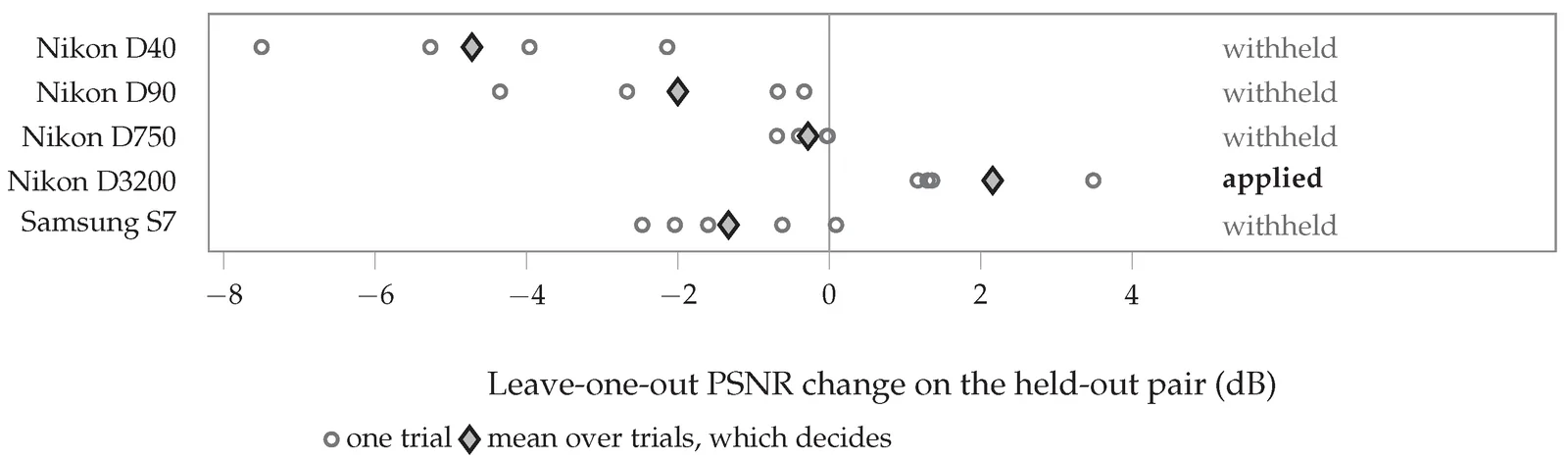
The calibration-only gate of Equation (6): leave-one-out PSNR change on held-out pairs, five k=3 trials per body, and the mean that decides. It reads no test image, needs k≥2, and applies the stage where the mean is positive.

***The calibration pairs decide when to add it.*** The correction is useful only if the body can be picked out in advance, without the measurement that identified it. Equation (6) does that from the calibration pairs alone: fit the stage on all but one pair, then score the change on the pair held out. It inspects no test image and consumes no test ground truth (Figure 8). Being a leave-one-out, it needs at least two pairs, in practice three. The stage can be fitted from one pair but not gated from one, and Figure 6 measures the cost. The decision separates the bodies as the measured outcomes do (Figure 8). The D3200 scores positive on all five trials, and the stage is applied; every control mean is negative, and it is not. Read at single-trial granularity, the rule can fire on a control, so the decision we validate is the five-trial mean. Figure 8 displays that budget: five trials of three pairs per body.

***The pairs also elect the denoiser.*** The denoiser gate of Section 3.2 keeps the bilateral filter on the D40 and the D3200 and elects non-local means on the D90, D750 and S7. On the full evaluation sets over five trials at k=3, the elected operator adds +0.99 dB and 0.21 of SSIM on the D750. On the S7, it adds +0.68 dB and 0.15. On the D40 and the D3200, the two operators lie within 0.18 dB of each other. On the D90 the elected operator scores 0.28 dB below the bilateral filter. It is the one body where the calibration-side choice and the test outcome differ.

### 4.3. Qualitative Analysis

**Figure 9:**
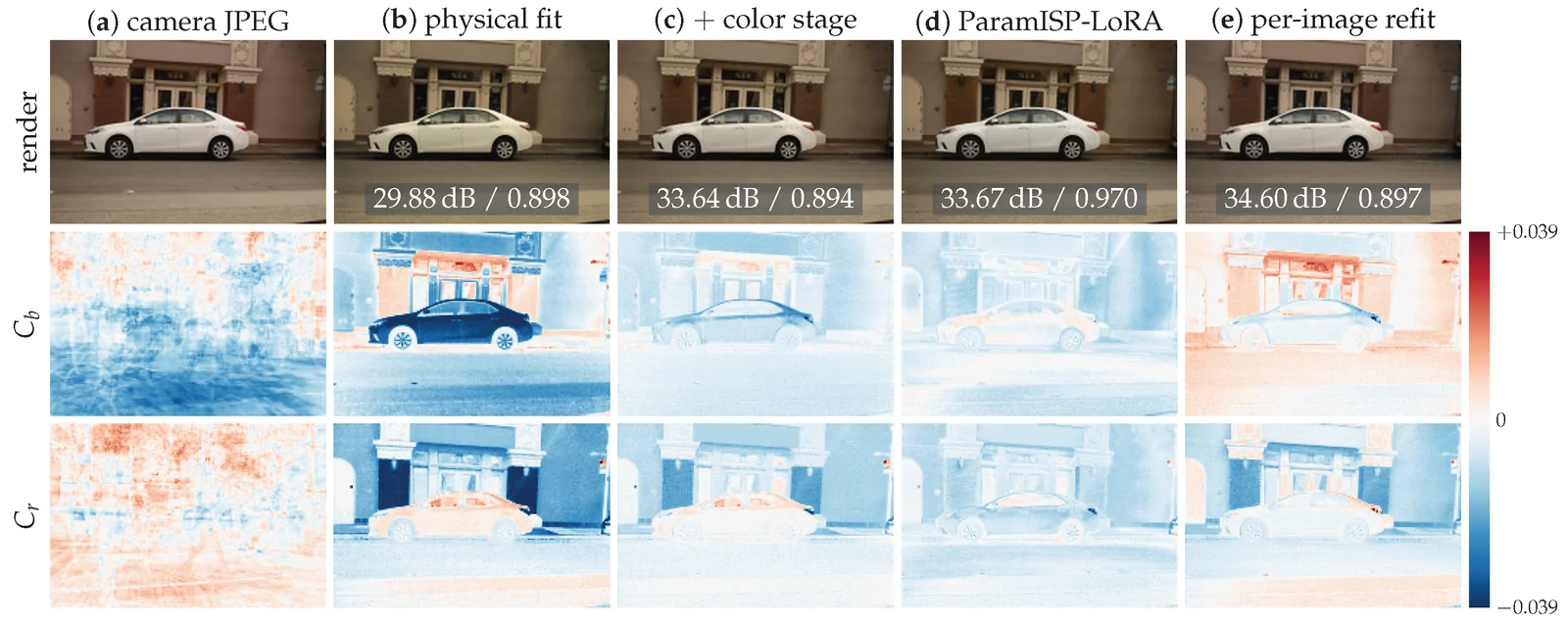
One held-out D3200 frame: renders (**top**) over signed $C_{b}$ and $C_{r}$ error maps, one shared bar; under (**a**,**b**)’s map averaged over 12 held-out frames. No evaluation target enters (**b**–**d**); the refit consumes this frame’s ground truth. Overlays: this frame’s PSNR/SSIM.

***What the correction removes on the D3200.*** Figure 9 sets five renders of one held-out D3200 frame over their signed chroma error maps. The maps are pooled to the large-scale drift that the band split of Section 5.2 separates from detail error. Averaged over the 12 held-out images, the stage takes chroma error RMS from 0.023 to 0.012 on the [0,1] sRGB scale. That is 73% of the energy over all bands. Across images rather than bands, the deployable fit’s drift is not a fixed offset. Over the 12 held-out frames beneath Figure 9a, its mean chroma error map carries only 16% of the per-image chroma error energy. Yet the color stage, consuming no test ground truth, removes 74% of the low-frequency chroma error energy on D3200 images outside its fit.

**Figure 10:**
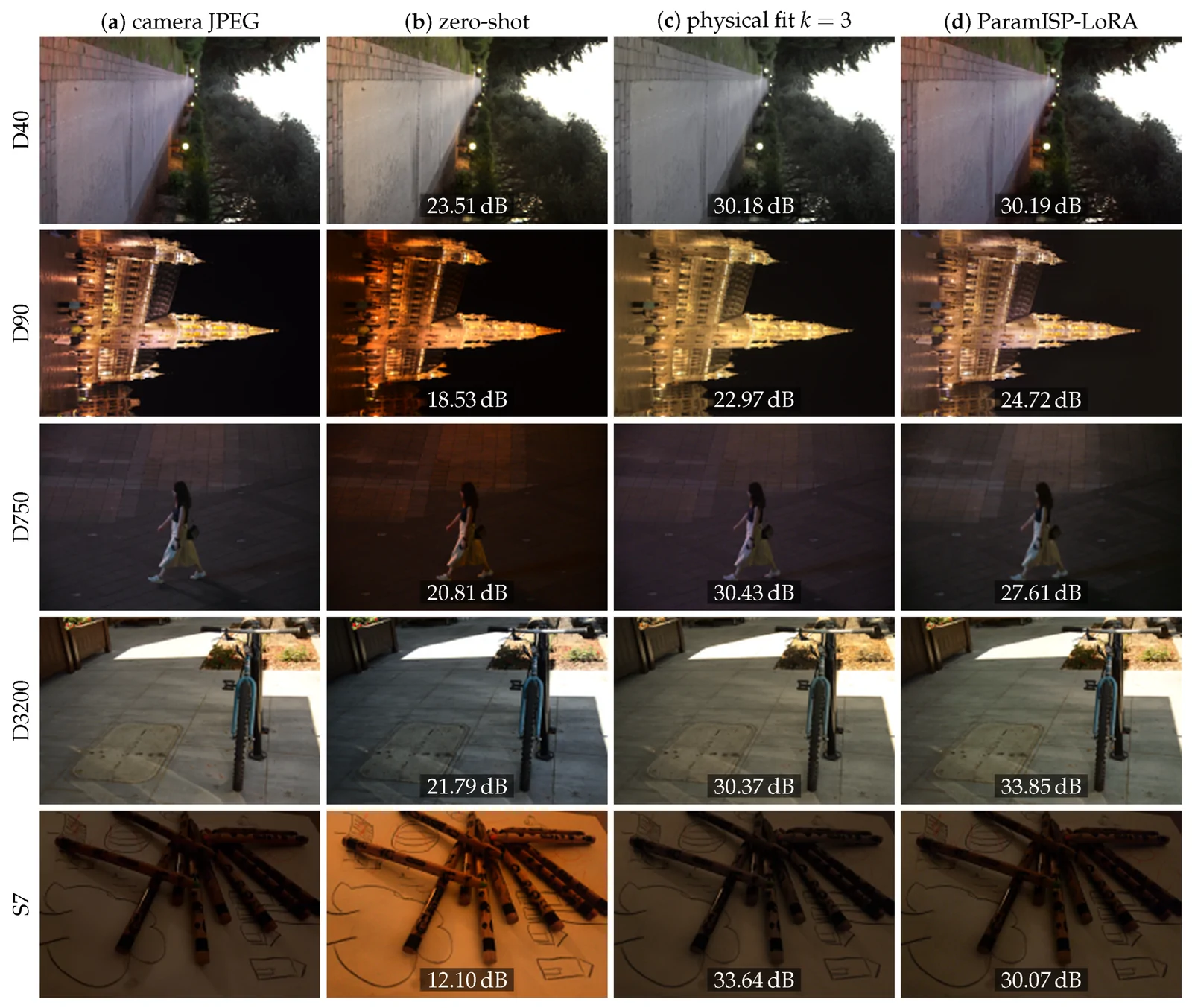
One held-out frame per body. Fit k=3, trial 0; S7 panels center-cropped to the shared 2:3 aspect; overlays are each frame’s own PSNR.

***What the fit gets wrong on five bodies.*** Figure 10 sets one held-out frame per body in four columns: the camera JPEG, the zero-shot render, the fit at three pairs, and the reference. The fit’s departures from the target are global casts of tone and color, not lost structure. The zero-shot cast, largest on the S7 and the D90, is removed by the fit; what remains is a global difference in brightness and saturation. Where the reference leads, it leads by the same global agreement (Section 4.2.1 and Section 5.6). The overlays are each frame’s own PSNR; the full-set numbers are those of Table 4.

## 5. Discussion

A margin is a property of the comparison at its resolution, not of a camera (Section 5.1). The D3200’s identification holds when the band edges, the sample and the search box change (Section 5.2). Every body shows the signature, and one body completes the sequence from prediction to gated correction (Section 5.3). The controller’s starting point is worth up to four decibels (Section 5.4). The calibration-time result holds beyond this pipeline (Section 5.5), and Section 5.6 states the scope and what remains after correction.

### 5.1. What the Margins Measure

***A margin is a property of the comparison.*** A margin says something about this comparison, not about the camera. Every decision in Figure 3b and Table 4 holds at the measured resolution of ±1.84 dB. On the D40, D90 and D750, the margins fall inside it, so those bodies leave the question open; closing it needs a finer comparison. On the S7, the refit passes the reference: settings of the seventy parameters that reach past it exist (Section 4.2.1). What constrains that body is the commitment to one θ per camera. The D90’s paired SSIM runs against the fit, with no band to read it against. Nothing constrains a mean-absolute-error refit to maximize a structural score. The D90 therefore remains a candidate second body where the reference leads, under a structural criterion, unresolved in either direction.

***Repeat-run reproducibility of the mechanisms.*** The physical fit reproduces exactly; the two deeper network fits do not. From the same pairs and seed, the physical fit repeats to 0.0000 dB and the tail decoder to 0.0019 dB. The full fine-tune of Table 3 falls into two calibration-loss modes across eleven repeats at one seed. Six of those repeats, scored on the pool, span 0.262 dB about the printed median. Four runs of the rank-one adapter span 0.198 dB and 12.3 percent of calibration loss. Both endpoints of the 2.67 dB class step therefore reproduce, while the two deeper mechanisms do not. A second calibration trial on the same pool returns the same ordering of the four mechanisms with a class step of 2.84 dB. The reference also moves 1.0–1.7 dB over one decade of its fitting rate and 0.90 dB across trials; these movements enter the resolution of Section 3.3.

### 5.2. What the Signature Identifies

**Figure 11:**
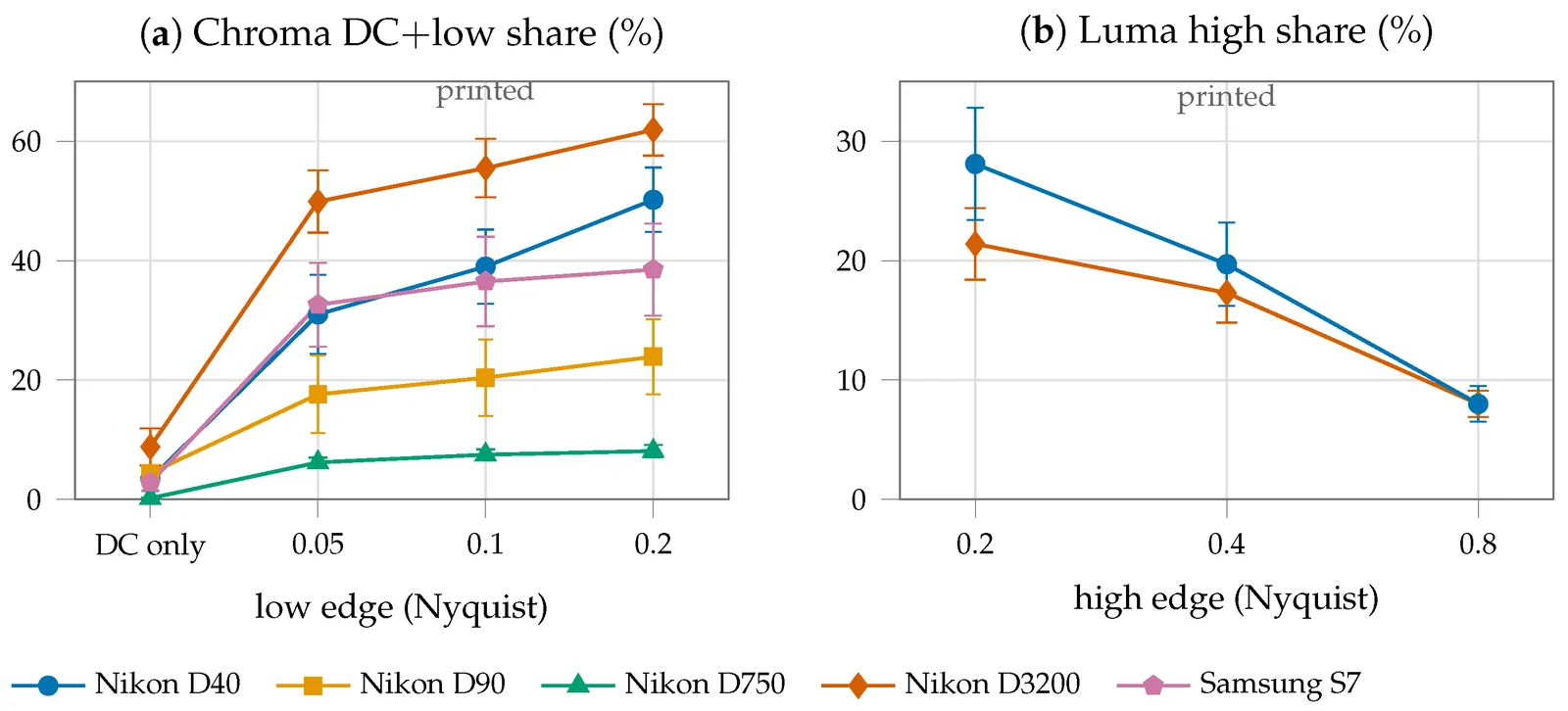
Band-cutoff sensitivity on 30 images per body, bars the 95% interval; printed setting 0.1/0.4. (**a**) Chroma DC+low share of the refit residual as the low edge moves; (**b**) luma-high share on the D3200 and the D40 as the high edge moves.

***Band cutoffs and sample size.*** The D3200’s rank depends neither on the band cutoffs nor on the sample. Figure 11 sweeps the low edge over {0.05,0.1,0.2} and the high edge over {0.2,0.4,0.8} of Nyquist. The D3200 ranks first, with its interval separated from the runner-up at every setting. The chroma DC+low share does not depend on the high edge, and a DC-only share collapses the separation. The printed bands are normalized radial frequencies: DC at f=0, low below 0.1 of Nyquist, mid from 0.1 to 0.4 and high from 0.4. Bayer color-difference aliasing lands in the high band and natural-scene chroma in the low band, so these edges separate the prediction. The 30 images per body are the first 30 frames in filename order, excluding the seed-0 calibration triple. A 95% interval accompanies each share, and the D3200’s gap to the runner-up is twice the summed intervals.

***Excess decomposition.*** Against the reference, the D3200’s extra error sits in chroma, where the derivation placed it. The excess error over the reference is measured on the same 30 D3200 images at the printed settings and decomposed along the ISP’s own division. The luma high band answers demosaicking, sharpening or denoising; low-frequency chroma answers white balance, color correction or tone. Chroma holds 89.20 ± 4.13% of the excess as residual power on the [0,1] sRGB scale. Its DC and low bands hold 77.24 ± 3.57% between them and the luma high band 17.31 ± 1.13%. The luma DC and low bands carry a negative share on every trial, with the refit being the more accurate render there. The refit falls behind in chroma and in the luma high band, the denoising choice occupies and leads in the lowest luma bands. This decomposition corroborates the reference-free signature of Section 4.2.1: the excess is the kind of error Section 3.1.1 predicts.

***Widening the search box.*** A wider search box does not close the gap. The refit drives the saturation gain to the top of its box on 386 of 500 D3200 images. Doubling that bound to 4.0 and refitting the same images gives +0.07 dB on the paired difference (95% interval [−0.13,+0.26]). SSIM moves by −0.0007, and the slot reaches the doubled ceiling on 454 images.

### 5.3. One Body Completes the Sequence

***Every body shows the signature.*** The signature ranks the D3200 first, but a share is not a size. Low-frequency chroma holds a share of every body’s refit residual, from 7.50% to 55.52% (Table 5). That share is largest, and separated from the runner-up, on the diagnosed body, though by absolute power the order differs. A share reads composition, not how far the limit is exceeded. Because the scene’s chroma spectrum enters both its numerator and its denominator, the order of shares across bodies is not separated from scene effects. The share is therefore corroboration.

***The shortfall splits in two.*** Only the D3200’s shortfall contains a gap the class cannot close. The shortfall against the reference at three pairs has two parts (Section 4.2.3). The class gap lies between the reference and the refit ceiling. The commitment gap, between the ceiling and the fit, is what one θ per camera costs. On the D40, D90, D750 and S7, the class gap lies inside the resolution, so they trail the reference through the commitment gap alone. That commitment gap is 0.39 dB on the D750 and 4.08 to 4.24 dB on the D40, D90 and S7. On the D3200, the class gap of 3.248 dB stands outside the resolution, and it is the operator Section 4.2.1 named. The within-body paired contrasts of Section 4.2.2 and Section 4.2.3 tie that body to the sequence. The named operator, fitted from the calibration pairs alone, recovers most of the gap, and the calibration-only gate admits it there alone.

***Gate criterion.*** The PSNR-only gate and a two-metric gate return the same decisions. The criterion of Equation (6) is PSNR. In Figure 6, the D750’s stage moves PSNR almost none and costs 0.016 of SSIM, a loss the rule does not score. The gate withholds the stage there regardless, so the loss does not occur. We tested a two-metric rule by re-rendering the held-out pairs of the same trials. It fires only where the held-out PSNR change is positive and the held-out SSIM change is non-negative. The rule fires on the D3200 and withholds on all four controls, as the PSNR rule does. No body separates the two rules on the trial mean. On the D3200, the held-out SSIM change is negative on two of five trials. The SSIM shortfalls on the D90 and the D750 remain under either rule.

### 5.4. What the Controller Contributes

**Figure 12:**
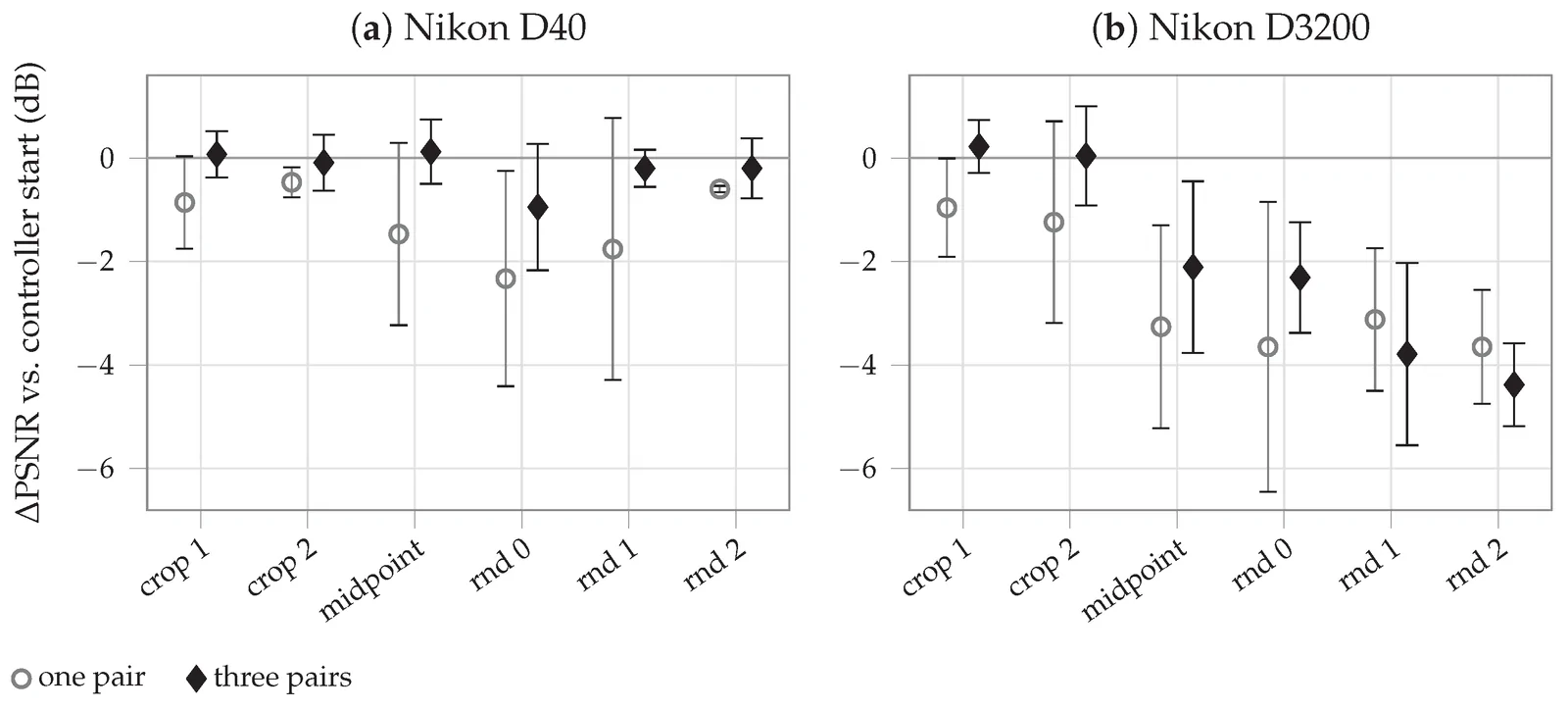
Held-out PSNR change relative to the controller start, same pairs and 200 steps, mean over five trials with standard deviation. Crop 1 and 2 repeat the controller start with another crop sequence. (**a**) Nikon D40; (**b**) Nikon D3200.

***Initialization at one and three pairs.*** The controller’s starting point is worth up to four decibels. The deployed inference path is network-free, but the fit is not. The controller’s one forward pass per body is part of what the fit’s quality rests on. From the same calibration pairs and seed, the physical fit reproduces to 0.0000 dB, so the starting point is the one remaining degree of freedom. Figure 12 refits both budgets on the D40 and the D3200 on the same five trials. The starts are the controller’s prediction, the midpoint of the boxes of Table 1, and three random points in the box. On the D40 at three pairs, the midpoint and two of the three random starts land within 0.2 dB of the controller start. At one pair, they fall 0.6 to 2.3 dB below it. On the D3200, every other start falls below the controller’s at both budgets. At one pair, the loss is 3.1 to 3.7 dB. At three pairs, it is 2.1 to 4.4 dB. Repeating the controller start with a different crop seed moves the one-pair fit by 0.5 to 1.2 dB. The three-pair fit moves by at most 0.2 dB. Its value is therefore between nothing and four decibels, depending on the body and the budget, and the controller supplies it.

***Step budget.*** On one D40 single-pair trial, the fit reaches 25.36 dB at 200 steps and 25.44 at 400; at 800, it stands at 24.24. A longer fit to one pair costs quality on the body’s other images.

### 5.5. Calibration Time Beyond This Pipeline

Calibration time follows how far back a gradient must travel, not how many parameters it updates. In Table 3, the rank-one adapter moves twenty-six times fewer parameters than the full fine-tune yet finishes only 0.8 percent sooner. Confining the fit to the last block is quicker than either (Section 4.2.2). A smaller adapted count reduces what an adaptation stores per body; it saves neither calibration nor inference time. The physical fit calibrates 2.8–5.5× faster than the adapter, with either denoiser, because no network stands in its gradient path. All cost ratios share one comparator, the 709,885-parameter base of Section 4.1.2, so each measures this pairing rather than weight-space adaptation as a class. That the deployed path runs no network does not depend on the comparator. We measured the distinction on one base network; the mechanism behind it, gradient depth, is not specific to ISPs.

### 5.6. Limitations

The scope is set by Table 2. Every controller and the reference descend from one training source, the Nikon D7000, with cross-source generality untested. Four of the five evaluation bodies are Nikon DSLRs of that make, with the Samsung S7 being the sole out-of-make body. Multi-frame, locally adaptive rendering, which is current smartphone practice, lies outside a class whose coefficients every pixel shares. The one mobile body here renders single-frame, so that practice is untested (Section 3.1.1). Every claim is limited to the tested class, single-frame Bayer pipelines: four Nikon DSLRs of the training make and one 2016 smartphone. Other color filter arrays and sensor technologies were not tested. The D3200’s frames were taken under one manual white balance (Table 2). PSNR, SSIM and LPIPS score imitation of one styled target—the camera’s own JPEG; no downstream consumer, detector or viewer, was tested on any render.

What survives the gated correction is unattributed. No test ground truth enters the procedure anywhere. At k=3, on the same 498 images and five trials, the corrected D3200 stands 1.90 dB below the reference in PSNR (Figure 4a). With the degree-three stage restored to the refit (Section 4.2.1), the refit stands 0.49 dB short in PSNR, inside the resolution, and 0.048 short in SSIM. That residual is likewise unattributed.

## 6. Conclusions

Because a controller predicts an ISP’s named physical parameters rather than weights, what the composition can express is derivable before any image is measured. Post-tone color freedom is one global scalar, so a hue-dependent transform there lies outside the class, with its absence predicted as low-frequency chroma error. Refitting the seventy parameters to each test image’s own ground truth tests that prediction. On one body of five, the Nikon D3200, the error appears in the predicted band and the refit falls 3.248 dB short of our weight-space reference. The measurement decides which body it is. Fitted in closed form from calibration pairs alone, the named stage recovers +3.28 ± 0.46 dB there against at most +0.48 dB on four controls. A calibration-only gate withholds it on all four. What still separates the corrected fit from the reference is unattributed by the remedies tested, yet attributable in principle. That is what naming the operators yields and an opaque weight tensor cannot. 

## Figures and Tables

**Table 1:** Layout of the 70-dimensional physical parameter vector θ, in the fixed execution order π used throughout. Ranges are the optimization box; its midpoint is one of the alternative starts of Section 5.4.

Stage	Slots	Quantity	Range
White balance	3	per-channel gains, green sites tied	[0.5,4.0]
Demosaic	46	scalars on the fixed GBTF kernel (identity =1)	[0.8,1.2]
Color correction	9	3 × 3 matrix, row-normalized	[−1.0,2.0]
Denoise	1	filter strength	[0.0,1.0]
Sharpen	2	unsharp mask: Gaussian σ, amount	[0,2], [0.5,3.0]
Tone	8	monotone piecewise-linear curve on *V*, softplus-mapped slots	[−2.0,2.0]
Saturation	1	global gain toward luminance	[0.5,2.0]
**Total**	**70**		

**Table 2:** Evaluation bodies. All targets are the camera’s own JPEG rendering, extracted from the RAW file.

Body	Source	Sensor	Capture notes	Images
Nikon D7000 (train)	RAISE [36]	16 MP	training source	5804
Nikon D40	RAISE [36]	6 MP	controls and mechanism studies	76
Nikon D90	RAISE [36]	12 MP	tagged Adobe RGB	2276
Nikon D750	NOD [37]	24 MP	night scenes	4218
Nikon D3200	PASCALRAW [38]	24 MP	manual white balance; one as-shot value on all frames	4259
Samsung S7	S7-ISP [39]	12 MP	mobile sensor	220

**Table 3:** Four adaptation mechanisms on one D90 trial of three pairs, scored on a disjoint 60-image pool. Network rows are our own retrains of ParamISP [2]; costs follow Section 4.1.3, CPU columns single-threaded.

	Fidelity		Parameters		Calibration (s)		Deployed		
Mechanism	PSNR	SSIM	Adapted	Resident	GPU	CPU	MAC/px	GPU ms	CPU s
ParamISP-FT, all weights	25.72	0.921	709,885	709,885	35.171	6848	273,161	1531	431
ParamISP-LoRA r=1, reference	24.52	0.897	27,481	737,366	34.893	5497	273,161	1531	431
ParamISP-tail, decoder	23.38	0.880	34,627	709,885	25.802	3943	273,161	1531	431
Ours, non-local means	20.71	0.739	70	70	12.245	1329	4925	372	81.1
Ours, bilateral	20.87	0.757	70	70	6.358	319	801	81.6	13.1

On the D90, the gate elects non-local means (Section 4.2.3).

**Table 4:** PSNR (dB) against the calibration budget: the physical fit (ours) and the reference at one, three and five pairs, and the per-image refit, which consumes test ground truth. Mean ± standard deviation over five trials; Δ is ours minus reference.

Body	Zero-	One Pair			Three Pairs			Five	Refit (Test GT)		Decision
	Shot	Ours	Ref.	Δ	Ours	Ref.	Δ	Ours	PSNR	Δ Ref. *k* = 3	
D40	19.45	24.72 ± 2.27	29.16 ± 0.78	−4.44	26.21 ± 0.74	30.46 ± 0.72	−4.25	26.35 ± 0.29	30.285	−0.179	within
D90	18.10	17.66 ± 1.85	21.20 ± 1.30	−3.54	20.78 ± 0.57	24.17 ± 0.73	−3.39	21.39 ± 0.18	24.953	+0.779	within
D750	22.18	23.78 ± 0.35	23.67 ± 0.44	+0.11	24.17 ± 0.15	24.53 ± 0.27	−0.36	24.14 ± 0.26	24.561	+0.029	within
D3200	22.72	28.12 ± 0.79	33.02 ± 0.60	−4.90	29.44 ± 1.08	34.62 ± 0.26	−5.18	30.26 ± 0.80	31.371	−3.248	fail
S7	14.50	23.28 ± 1.36	23.64 ± 1.68	−0.36	23.62 ± 0.83	24.45 ± 0.90	−0.83	24.40 ± 0.81	27.855	+3.403	pass

**Table 5:** Refit residual power shares (%) in luma/chroma × DC/low/mid/high on 30 images per body, with 95% intervals; PSNR is the refit’s subset mean. Every column consumes test ground truth. Band cutoffs: Section 5.2.

Body	Refit	Share of That Body’s Own Refit Residual (%)			Rank by abs.
	PSNR (dB)	Chroma	Chroma DC+Low	Luma High	DC+Low Power
Nikon D40	30.351	60.52	38.97 ± 6.17	19.65	5
Nikon D90	25.330	26.97	20.37 ± 6.44	12.40	1
Nikon D750	22.411	8.36	7.50 ± 0.95	0.75	2
Nikon D3200	31.454	70.37	55.52 ± 4.87	17.32	4
Samsung S7	28.316	39.43	36.54 ± 7.50	14.41	3

## Data Availability

The evaluation data are the public RAISE [36], NOD [37], PASCALRAW [38] and S7-ISP [39] datasets (Table 2). The project repository is https://github.com/jinook27/single-pair-isp-calibration (accessed on 12 October 2026).
